# Neuropathological comparisons of amnestic and nonamnestic mild cognitive impairment

**DOI:** 10.1186/s12883-015-0403-4

**Published:** 2015-08-20

**Authors:** Brittany N. Dugger, Kathryn Davis, Michael Malek-Ahmadi, Joseph G. Hentz, Shawn Sandhu, Thomas G. Beach, Charles H. Adler, Richard J. Caselli, Travis A. Johnson, Geidy E. Serrano, Holly A. Shill, Christine Belden, Erika Driver-Dunckley, John N. Caviness, Lucia I. Sue, Sandra Jacobson, Jessica Powell, Marwan N. Sabbagh

**Affiliations:** Civin Laboratory for Neuropathology, Banner Sun Health Research Institute, 10515 W. Santa Fe Dr., Sun City, AZ 85351 USA; The Cleo Roberts Center for Clinical Research, Banner Sun Health Research Institute, Sun City, AZ USA; Mayo Clinic, Scottsdale, AZ USA

## Abstract

**Background:**

Although there are studies investigating the pathologic origins of mild cognitive impairment (MCI), they have revolved around comparisons to normal elderly individuals or those with Alzheimer’s disease (AD) or other dementias. There are few studies directly comparing the comprehensive neuropathology of amnestic (aMCI) and nonamnestic (naMCI) MCI.

**Methods:**

The database of the Brain and Body Donation Program (www.brainandbodydonationprogram.org), a longitudinal clinicopathological study of normal aging and neurodegenerative disorders, was queried for subjects who were carrying a diagnosis of aMCI or naMCI at the time of autopsy. Neuropathological lesions, including neuritic plaques, neurofibrillary tangles (NFTs), Lewy bodies (LBs), infarcts, cerebral white matter rarefaction (CWMR), cerebral amyloid angiopathy (CAA), and concurrent major clinicopathological diagnoses, including Parkinson’s disease (PD) were analyzed.

**Results:**

Thirty four subjects with aMCI and 15 naMCI met study criteria. Subjects with aMCI were older at death (88 vs. 83 years of age, *p* = 0.03). Individuals with naMCI had higher densities of LBs within the temporal lobe (*p* = 0.04) while subjects with aMCI had a propensity for increased NFTs in parietal and temporal lobes (*p* values = 0.07). After adjusting for age at death, the only significant difference was greater densities of temporal lobe NFTs within the aMCI group. Other regional pathology scores for plaques, NFTs, and LBs were similar between groups. Subjects met clinico-pathological criteria for co-existent PD in 24 % aMCI and 47 % naMCI while neuropathological criteria for AD were met in equal percentages of aMCI and of naMCI cases (53 %); these proportional differences were not significant (*p* values > 0.35). Furthermore, regardless of amnestic status, there was a greater presence of CAA (71 % of MCI with executive dysfunction vs. 39 % without *p* = 0.03) and a greater presence of CWMR (81 % of MCI with executive dysfunction and 54 % without *p* = 0.046) in MCI cases with executive dysfunction.

**Conclusions:**

No single pathologic entity strongly dichotomized MCI groups, perhaps due to the pathologic heterogeneity found within both entities. However, these data suggest the possibility for naMCI to have a propensity for increased LBs and aMCI for increased NFTs in select anatomic regions.

## Background

Mild cognitive impairment (MCI) is a clinical diagnostic term for elderly subjects with defined forms of cognitive dysfunction that do not meet criteria for dementia [1]. In most subjects, MCI is an intermediate phase between normal cognition and dementia. The term was first used nearly a quarter of a century ago by Reisberg and colleagues, who observed a progression to dementia in an elderly population with mildly impaired cognitive function [2]. Thirteen years later, the classification of MCI as proposed by the 2^nd^ International Working Group on MCI Criteria included the subtypes of amnestic MCI (aMCI) and nonamnestic MCI (naMCI) [1, 3]. Memory impairment is the defining feature of aMCI, while naMCI is defined by deficits of other cognitive abilities, including attention, executive function, visuospatial skills, and language. Both aMCI and naMCI may affect single or multiple neuropsychological domains. In 2011, criteria were published focusing on MCI due to Alzheimer's disease (AD) [4].

Most studies investigating the pathologic origins of MCI have revolved around comparisons to normal elderly individuals or those with AD or other dementias [5–13]. There are few studies directly comparing the comprehensive neuropathology of aMCI and naMCI. It is critical to investigate pathological similarities and differences between aMCI and naMCI given the proposed dichotomous progression of each of these diagnoses- i.e. aMCI progressing more towards AD [9, 12]. The purpose of this study is to compare and contrast common neuropathologies in subjects who were diagnosed with aMCI or naMCI.

## Methods

### Subjects

Subjects were selected from the Arizona Study of Aging and Neurodegenerative Disorders (AZSAND) with autopsies in the Brain and Body Donation Program (BBDP) at Banner Sun Health Research Institute in Sun City, Arizona (www.brainandbodydonationprogram.org) [14, 15]. All enrolled subjects or their legal representatives sign a written informed consent approved by an Institutional Review Board (Western Institutional Review Board, Puyallup, WA) before the time of death allowing both clinical assessments during life and several options for brain and/or body organ donation after death. The Institutional Review Board approval covers any studies conducted with data and/or tissues from deceased BBDP subjects. Permission to access the research database with HIPPA compliant de-identified information was granted by the director of the BBDP. Access to the research database is open to investigators who are part of the Arizona Alzheimer’s Disease Consortium, the Arizona Parkinson’s Disease Consortium, as well as researchers who are registered on the BBDP website (www.brainandbodydonationprogram.org) all of which are approved to access the database under the Institutional Review Board. Annual visits and exams for cognition and movement disorders and criteria for clinical diagnoses are previously described [14]. All assessments were conducted in accordance with the Declaration of Helsinki.

The BBDP database was queried for individuals who were autopsied between January 1997 through July 2014, had at least one formal, standardized BBDP neurological evaluation during life, were carrying a diagnosis of aMCI or naMCI according to published criteria [1, 4] at their last visit prior to death, and had a completed neuropathological evaluation. Cases were excluded if they had a diagnosis of dementia or had been thought to have cognitive dysfunction that was secondary to a medical illness. If an individual was not seen within 18 months before death, a standardized telephone interview was conducted with a knowledgeable contact, typically next of kin, to evaluate for the presence of dementia or a movement disorder in the final year of life. Additionally, at least two years of private medical records were available for each subject and these were reviewed to provide supplementary information where necessary.

### Clinical assessment

Prior to death, all participants were assessed annually on a variety of clinical, neuropsychological, and functional measures as described previously [14]. Cognition was assessed using an array of measures, which are detailed in a recent publication of the BBDP [15]. A supervising geriatric psychiatrist and neuropsychologist (SJ and CB, respectively) at the Banner Sun Health Research Institute oversaw administration of these measures. Results of these assessments were jointly interpreted by a geriatric psychiatrist (SJ), behavioral neurologist (MNS, RJC), movement disorders neurologist (CHA, HAS, JNC, ED), and neuropsychologist (CB) in order to reach a consensus diagnosis for each individual. With respect to initial disagreements among the consensus panel, all collateral information available (previous years’ assessments, medical records, non-cognitive questionnaires, mood measures, functional assessments, etc.) is reviewed and discussed until a consensus is reached. For the clinical diagnoses of aMCI and naMCI, published criteria were used in which neuropsychological test performance fell at or below 1.5 standard deviations in one or more domains of cognition [1, 4].

### Neuropathological assessment

All cases underwent autopsy and had a standardized neuropathological assessment blinded to clinical categorization. The final clinicopathological diagnoses were based on both the neuropathology and clinical characteristics as obtained from standardized neurological assessments as well as review of private medical records. Criteria for these diagnoses have been reviewed elsewhere [16, 17]. A clinicopathological diagnosis of Parkinson's disease (PD) was made if the subject had two or more cardinal clinical signs (rest tremor, bradykinesia, rigidity) as well as Lewy bodies and pigmented neuron loss in the substantia nigra. All subjects were genotyped for apolipoprotein E (ApoE) using a modification of a standard method [18].

Tissue processing methods have been previously described [14, 19]. In brief, formaldehyde-fixed 5 μm paraffin-embedded sections were stained with hematoxylin and eosin, while large-format, 40–80 μm-thick formaldehyde-fixed sections were stained for plaques, tangles and other features using Gallyas, Thioflavin-S and Campbell-Switzer methods [20]. All subjects were classified according to the Unified Staging System for Lewy Body (LB) Disorders [21] after immunohistochemical evaluation of phosphorylated α-synuclein on 5 μm paraffin sections [22]. Individuals with incidental LBs (iLBs) were defined as those lacking signs of parkinsonism and/or dementia during life but with autopsy findings of positive phosphorylated α-synuclein-immunoreactive neuronal elements, morphologically consistent with Lewy-type pathologies. Semi-quantitative analyses of neuritic plaques (NP) and neurofibrillary tangle (NFT) densities were done using standardized Consortium to Establish a Registry for Alzheimer’s Disease (CERAD) published templates [23]; estimates of none, sparse, moderate, or frequent were converted to a continuous 0-3 scale for statistical purposes. Regions scored included cortical gray matter from standard levels of frontal, parietal, and temporal lobes, as well as hippocampal and entorhinal regions. Global NP and NFT scores were obtained by summation of all regions analyzed with a maximum score of 15. For this study, subjects received a pathological diagnosis of AD if they were classified as “intermediate” or “high” according to the NIA-Reagan criteria, regardless of the absence of dementia [24]; Braak NFT stage was also determined [25]. Cerebral white matter rarefaction (CWMR) was defined as having 26 % or more of the centrum semiovale affected in one or more of the following lobes: frontal, parietal, occipital, and temporal; these methods have been published previously [26]. Cerebral amyloid angiopathy (CAA) was graded by density of amyloidotic blood vessels, using a 0–3 scale analogous to CERAD templates [23]. Brain neoplasms were defined as primary or metastatic parenchymal tumors and did not include meningiomas or schwannomas. Argyrophilic grains (Arg) were defined as typical spindle-shaped structures revealed by the Gallyas silver stain [27]. Presence/absence of non-acute infarcts were categorized by affected region (cortical, centrum semiovale, deep nuclei, and/or infratentorial regions) and by size (microscopic (not grossly visible), lacunar (<1 cc), small (1–27 cc), and large (>27 cc)). Infarct groups were not mutually exclusive. For evaluation of mixed pathologies- only the three diagnoses of 1) meeting neuropatholgic criteria for AD; 2) cases having LBs in areas other than the olfactory bulb, and 3) presence of non-acute infarcts were considered. These were then divided into cases with none, one, two, or three of the mentioned diagnosis; similar methods have been done previously [28].

### Statistical analysis

Two-tailed, unpaired *t* tests were used to compare differences between aMCI and naMCI cases on age at death, time from MCI diagnosis to death, years of education, Mini Mental State Examination (MMSE) score and Part III (motor) of the Unified Parkinson’s Disease Rating Scale (UPDRS). Pearson chi-square tests were used to determine differences in frequency for neuropathological diagnosis of AD, concurrent major clinicopathological diagnoses, Arg, CAA, CWMR, brain neoplasms, iLBs, infarcts, gender, and ApoE ε4 carrier status. The Fisher exact test was used instead of the Pearson chi-square test when the minimum expected cell count was less than five. The Wilcoxon rank-sum test was used to compare Braak stage, NP score, and Unified Lewy Body stage. After initial analyses, all data were then adjusted for age at death.

## Results

### Overall demographics

Of the 1185 autopsied BBDP subjects queried, 49 (4 %) met inclusion criteria for aMCI or naMCI. Of these 49 subjects, 20 (40 %) were female, mean (±SD) age at death was 87 ± 7.8 years (range 69–103 years), and the average time from last neurological evaluation to death was 14 ± 13.3 months (range 2 to 65 months). Thirty-four (69 %) met criteria for aMCI, while 15 (31 %) were naMCI.

### Differences and similarities between aMCI and naMCI

Demographic and pathologic characteristics of the two groups are displayed in Table 1. The aMCI group was significantly older, but there were no large differences with respect to gender ratios, ApoE4 carrier frequency (there were no aMCI or naMCI that were homozygous for ApoE4), time from last neurological (neuro) exam until death, last MMSE score and last UPDRS score. With respect to domains affected, naMCI has significantly greater frequencies of cases with executive dysfunction (67 vs. 32 %, *p* = 0.03). There were no other large differences with respect to demographic information between aMCI and naMCI (differences less than 22 percentage points or 0.4 standard deviations).

**Table 1:** Demographic information of aMCI and naMCI cases in the BBDP. Mean ± SD or *n* (%)

	aMCI (*N* = 34)	naMCI (*n* = 15)	95 % CI	*P* value
Female (%)	12 (35 %)	7 (47 %)	−0.41 to 0.19	0.53
Multi-domain *N* (%)	13 (38 %)	4 (27 %)	−0.16 to 0.39	0.43
Domains affected				
Memory	34 (100 %)	n/a	n/a	n/a
Executive	11 (32 %)	10 (67 %)	−0.63 to −0.06	0.03
Language	4 (12 %)	5 (33 %)	−0.50 to 0.03	0.11
Visuospatial	2 (6 %)	3 (20 %)	−0.42 to 0.06	0.16
Attention	1 (3 %)	2 (13 %)	−0.36 to 0.07	0.22
Age at death, yrs	88 ± 8	83 ± 7	0.4 to 9.8	0.03
Interval last neuro exam till death, months	15 ± 15	11 ± 7.7	−0.61 to 0.67	0.86
Education, yrs.	15 ± 3	16 ± 2	−3.3 to 0.0	0.05
APOE 4 carriers	9 (26 %)	6 (40 %)	−0.43 to 0.15	0.50
Last MMSE	27 ± 2	27 ± 2 [*n* = 14]	−1.8 to 0.9	0.55
Interval last MMSE till death, months	15 ± 15	10 ± 5 [*n* = 14]	−3.3 to 13.2	0.23
Last UPDRS	20 ± 16 [n = 33]	23 ± 17	−13 to 8	0.63
Interval last UPDRS till death, months	15 ± 15	17 ± 19	−12 to 9	0.78

Some subjects had concomitant clinicopathological diagnoses (Table 2), including Parkinson’s disease (PD), progressive supranuclear palsy (PSP), motor neuron disease (MND), and multiple system atrophy (MSA); frequencies of these diagnoses were not substantially different between aMCI and naMCI (differences less than 22 percentage points). Subjects met clinicopathological criteria for co-existent PD in 24 % aMCI and 47 % naMCI while neuropathological criteria for AD were met in equal percentages of aMCI and of naMCI cases (53 %).

**Table 2:** Frequencies of pathologies within aMCI (*N* = 34) and naMCI (*N* = 15) cases. All pathology groups are not mutually exclusive; there is considerable overlap with concomitant pathologies

	aMCI	naMCI	*p* value
Clinicopathologic diagnoses			
PD	8 (24 %)^a^	7 (47 %)	0.18
PSP	3 (9 %)^a^	3 (20 %)	0.35
MND	0	1 (7 %)	0.31
MSA	1 (3 %)	0	1.00
Other pathologies			
Met neuropath criteria for AD	18 (53 %)	8 (53 %)	1.00
Braak NFT stage			0.22
I	2 (6 %)	0	
II	2 (6 %)	2 (13 %)	
III	3 (9 %)	5 (33 %)	
IV	23 (68 %)	8 (53 %)	
V	3 (9 %)	0	
VI	1 (3 %)	0	
CERAD NP score			0.90
None	12 (35 %)	6 (40 %)	
Sparse	2 (6 %)	1 (7 %)	
Moderate	8 (24 %)	2 (13 %)	
Frequent	12 (35 %)	6 (40 %)	
Incidental LBs	5 (15 %)	0	0.31
Unified LB staging scheme			0.18
Stage 0. no LBs	21 (62 %)	8 (53 %)	
Stage 1. OBT only	2 (6 %)	0	
Stage IIa. Brainstem	3 (9 %)	1 (7 %)	
Stage IIb. Limbic	3 (9 %)	0	
Stage III. Limbic + Brainstem	3 (9 %)	3 (20 %)	
Stage IV. Neocortical	2 (6 %)	3 (20 %)	

^a^One aMCI case had both PSP and PD; there were no other overlapping clinicopathological diagnoses

All MCI cases had one or more of the following pathologies: neurofibrillary tangles (NFTs), NP, and LBs– the most common being NFTs. All aMCI and naMCI contained at least some NFTs, while 12 (35 %) aMCI and 6 (40 %) naMCI were devoid of NP in all areas examined; categories of age related CERAD NP density scores and Braak NFT staging are listed in Table 2. Both aMCI and naMCI had similar distributions and densities of NP (Table 3). However, for NFTs, there was a propensity for aMCI to have higher densities than naMCI in the temporal and parietal lobe (Bayes posterior probability 96 %), although this did not reach statistical significance (*P* values = 0.07). For the presence of Lewy-type alpha-synucleinopathy (Table 3), only the temporal lobe was statistically significant (*p* = 0.04) with naMCI having greater densities than aMCI. With respect to overall differences in LBs (i.e. examining just for the presence of LBs irrespective of any parkinsonian features), there were no large differences based on the presence of LBs, with 38 % of aMCI and 43 % of naMCI having LBs (95 % CI: -0.36 to 0.26 *p* = 0.73). After adjusting for age at death, the only large difference was greater temporal lobe NFTs within the aMCI group (95 % CI: 0.00 to 0.69, *p* = 0.05). As for other pathologies, the most common pathologies in aMCI and naMCI were CWMR (65 % aMCI and 67 % naMCI) and CAA (67 % aMCI and 47 % naMCI). There were only 2 cases with brain neoplasms both of which had aMCI. There were also similar frequencies with respect to Arg (37 % aMCI, 27 % naMCI) and acute infarcts (18 % aMCI, 13 % naMCI). Frequencies for non-acute infarcts are located in Table 4. The frequency for non-acute infarcts for aMCI was 53 and 40 % for naMCI. There were no significant differences on any infarct measures (location and/or size) between aMCI and naMCI. No case had a pathological diagnosis of hippocampal sclerosis.

**Table 3:** Semi-quantitative analyses of neuritic plaques (NP) and neurofibrillary tangles (NFT), Lewy-type synucleinopathy (listed as average (mean)), and other pathologies (listed as frequencies) within aMCI (*N* = 34) and naMCI (*N* = 15)

	aMCI	naMCI	95 % CI	*p*-value
Neuritic plaques				
Frontal lobe	1.5 (1.3)	1.8 (1.3)	−1.0 to 0.6	0.55
Temporal lobe	1.5 (1.3)	1.5 (1.3)	−0.7 to 0.9	0.88
Parietal lobe	1.6 (1.3)	1.7 (1.7)	−1.0 to 0.7	0.76
Hippocampus region	0.7 (0.9)	0.7 (0.8)	−0.53 to 0.53	1.00
Entorhinal region	1.3 (1.3)	1.3 (1.0)	−0.8 to 0.8	0.97
Total (all areas; score out of 15)	6.6 (5.7)	1.2 (5.2)	−4.3 to 2.9	0.11
Neurofibrillary tangles				
Braak NFT stage	IV (I-VI)	III (II-IV)	−0.22 to 0.95	0.22
Frontal lobe	0.4 (0.6)	0.2 (0.3)	−0.06 to 0.60	0.11
Temporal lobe	1 (0.8)	0.6 (0.7)	−0.04 to 0.91	0.07
Parietal lobe	0.4 (0.6)	0.1 (0.2)	−0.02 to 0.60	0.07
Hippocampus region	2.2 (0.9)	2.0 (0.9)	−0.30 to 0.80	0.36
Entorhinal region	2.6 (0.8)	2.5 (0.8)	−0.38 to 0.60	0.66
Total (all areas; score out of 15)	6.6 (2.8)	5.2 (2.3)	−0.3 to3.1	0.11
Lewy type synucleinopathy				
Olfactory bulb	1.1 (1.6)	1.5 (1.8)	−1.5 to 0.7	0.44
Cingulate cortex	0.6 (1.1)	1.2 (1.6)	−1.4 to 0.1	0.11
Frontal lobe	0.2 (0.5)	0.4 (0.8)	−0.55 to 0.22	0.39
Temporal lobe	0.2 (0.5)	0.7 (1.0)	−0.9 to -0.3	0.04
Parietal lobe	0.2 (0.5)	0.5 (0.7)	−0.64 to 0.12	0.17
Amygdala	0.9 (1.5)	1.5 (1.8)	−1.5 to 0.5	0.29
Transentorhinal cortex	0.6 (1.1)	1.1 (1.5)	−1.3 to 0.3	0.23
Total (all areas; score out of 15)	6.6 (2.8)	5.2 (2.3)	−0.3 to3.1	0.11
Other pathologies				
Arg	13 (37 %)	4 (27 %)	n/a	0.74
CWMR	22 (65 %)	10 (67 %)	n/a	0.89
CAA	16 (47 %)	10 (67 %)	n/a	0.21
Brain neoplasms	2 (6 %)	0	n/a	1.0
Acute infarcts	6 (18 %)	2 (13 %)	n/a	1.0

**Table 4:** Infarcts (excluding acute) in aMCI and naMCI organized by location and size (microscopic (not grossly visible), lacunar (<1 cc), small (1-27 cc), and large (>27 cc)). All data listed as N (%). Groups are not mutually exclusive

	aMCI				
Area affected	Total cases	Microscopic	Lacunar	Small	Large
Cortical	10 (29 %)	8 (24 %)	2 (6 %)	2 (6 %)	2 (6 %)
Centrum semiovale	3 (9 %)	2 (6 %)	1 (3 %)	1 (3 %)	1 (3 %)
Deep nuclei	12 (35 %)	4 (12 %)	9 (26 %)	2 (6 %)	1 (3 %)
Infratentorial	14 (41 %)	12 (35 %)	2 (6 %)	2 (6 %)	1 (3 %)
	naMCI				
Area affected	Total cases	Microscopic	Lacunar	Small	Large
Cortical	3 (20 %)	1 (7 %)	0	2 (13 %)	1 (7 %)
Centrum semiovale	3 (20 %)	0	2 (13 %)	0	1 (7 %)
Deep nuclei	3 (20 %)	2 (13 %)	2 (13 %)	0	0
Infratentorial	3 (20 %)	2 (13 %)	1 (7 %)	0	0

**Fig. 1:**
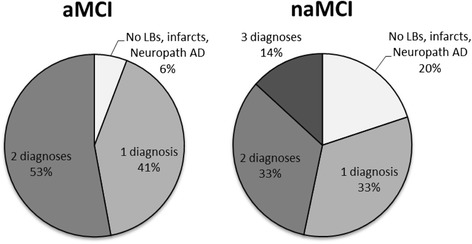
Pie charts depicting the frequencies of the number of mixed pathological diagnoses (infarcts, LBs, and neuropathological diagnosis of AD) for aMCI and naMCI

With respect to mixed pathologies (infarcts, any LBs, and/or a neuropathologic diagnosis of AD), 20 % of naMCI and 5.9 % of aMCI contained none of the pathologies (*p* = 0.16, Fisher’s Exact Test), 33.3 % of naMCI and 41.2 % of aMCI contained 1 of the pathologies (χ^2^ = 0.04 *p* = 0.84), 33.3 % of naMCI and 52.9 % of aMCI contained at 2 of the pathologies (χ^2^ = 0.92 p = 0.34), and 13 % of naMCI contained all three (*p* = 0.09, Fisher’s Exact Test) (Fig. 1).

Lastly, with respect to the presence/absence of executive dysfunction (regardless of aMCI or naMCI status) there were some significant differences. There was a significant relationship between the presence of CAA and executive dysfunction, (71 % of MCI with executive dysfunction and 39 % without executive dysfunction- 95 % CI: 0.06 to 0.59 *p* = 0.03) and also a significant relationship between the presence of CMWR and executive dysfunction (81 % of MCI with executive dysfunction and 54 % without - 95 % CI: 0.02 to 0.52 *p* = 0.046).

## Discussion

The current study demonstrates the vast array of neuropathologic heterogeneity within MCI, suggesting that there may be no single underlying etiology dichotomizing aMCI from naMCI. This is supported by previous studies demonstrating multiple pathologies are found to be present in MCI [5, 6, 10, 29]. This study differs from most previous studies by directly comparing neuropathology in two main subtypes of MCI, i.e. aMCI and naMCI. Despite heterogeneity, MCI subtypes did have propensities for certain demographic and pathological measures. Individuals who came to autopsy with a diagnosis of aMCI during life were older at death, and were less likely to have executive dysfunction when compared to naMCI. Furthermore, amongst the plethora of pathologies, naMCI had a propensity for increased LBs and aMCI increased NFTs in select anatomic regions.

Although there have been an increased number of studies investigating the pathologic origins of MCI, these have mostly revolved around comparisons to cognitively normal elderly or those with AD or other dementias [5–13] with few studies directly comparing neuropathological differences between aMCI and naMCI. One of the best studies on this comparison was conducted through the Religious Orders Study/Rush Memory and Aging Project (ROS/MAP) which examined LBs, and AD pathologies in 75 individuals who died with aMCI and 59 with naMCI [10]. Although the ROS/MAP study did not describe anatomic distributions of LBs, AD pathologies, CAA, CWMR, or Arg, they did demonstrate that 59 % of aMCI met pathological criteria for the diagnosis of AD compared to 49 % of naMCI; these percentages are very similar to those presented in the current study. The ROS/MAP study also had similar conclusion that many individuals with MCI exhibit mixed pathologies, thus emphasizing pathological heterogeneity.

In this cross-sectional study, although no global differences in AD pathology were found (i.e. meeting pathological criteria for AD), certain anatomic regions (parietal and temporal lobes) had a propensity for increased NFTs in subjects with aMCI (p values = 0.07). Furthermore, in both MCI groups, no cases were completely devoid of NFTs. In contrast, 26/50 (52 %) of MCI cases were devoid of NPs. Given temporal lobe structures are affected early within the pathologic course of AD (as determined by Braak NFT stages) [25], this is in support of clinical studies having shown aMCI to have a greater conversion rate to probable AD than naMCI [29, 30]. When examining MCI as one entity, several studies have described the presence of increased numbers of NFTs in subjects with MCI as compared with similarly-aged normal subjects, especially in medial temporal lobe structures [30, 31]. Others have examined similarities in pathology between aMCI and AD, suggesting that aMCI is identical to early AD based on NFT distribution [7, 9].

As for other neuropathological comparisons, a recent pathologic study demonstrated naMCI to have a greater conversion rate to dementia with LBs by time of autopsy [12]. With respect to PD, in a multicenter, pooled, clinically based study examining 347 PD with MCI, 49 % had naMCI and 51 % had aMCI [32]. Other data from the BBDP published on MCI within the setting of PD, too small to correlate pathologic and clinical findings, revealed varying LBs distribution [29]. In the current study, there was no large difference in the concurrency rate of clinicopathologically defined PD; however, Lewy-type alpha-synucleinopathy had greater densities in the temporal lobe of naMCI. This distinct anatomic difference may be due to the temporal lobe (similar to data on NFTs) being affected somewhat early in the Lewy-type alpha-synuclein disease process [21].

Other pathologies, outside the realm of major clinicopathologically-defined conditions, such as CWMR, Arg, and CAA, may exist within MCI as well as within clinically normal elderly individuals. The most common pathology within the aMCI and naMCI groups were CWMR and CAA and both were equally common, although reports have demonstrated that CWMR and CAA have a greater association with AD [33–36]. Although no large associations were found with respect to amnestic type, there were associations with the executive dysfunction. Cases with executive dysfunction had larger frequencies of CWMR and CAA. A radiological study of white matter lesions in 129 patients demonstrated that the severity of white matter lesions was not associated with time to conversion to dementia for either MCI patients in general or amnestic MCI patients in particular [37]. Our frequencies of Arg in each subgroup are very similar to those that have been previously reported for the entire MCI entity [5].

One of the difficulties in conducting pathologic studies on MCI patients after death is that few autopsies have been done on individuals who died while still in the MCI stage; this is evident in the current study with less than 5 % of the total number of BBDP participants having died in the MCI stage. Larger samples would be needed in order to detect or rule out smaller subtle differences between aMCI and naMCI. This study further emphasizes the dire need for in-vivo biomarkers that could demonstrate disease progression of LBs and NFTs, thus providing firmer conclusions. Individuals who die and undergo autopsy while in the MCI state may not represent the full spectrum of MCI due to survival bias, reversion back to normal cognition, and those who converted to dementia before death. One report revealed the mean time to developing dementia from MCI baseline was 3.1 years for those eventually diagnosed as clinically probable AD, while this was 2.6 years for those eventually diagnosed with dementia with Lewy bodies [12]. Furthermore, cases included in this study were mainly elderly volunteers, typically over the age of 70 at enrollment. Due to the emphasis of the program being the study of normal aging, PD, as well as dementia there is a recruitment bias towards enrolling subjects in these categories. Hence there may be an overrepresentation of Parkinsonian diagnoses in aMCI and naMCI- although no differences were seen between groups. Although this study had limitations, such as the small sample size and the greater proportion of aMCI cases, perhaps due to the known prevalence of MCI subtypes [6, 38], it is the most comprehensive study to date analyzing a full range of pathologies among amnestic and nonamnestic MCI subjects.

## Conclusions

This small study suggests there may be no single underlying entity strongly associated with a specific domain deficit within MCI, given the finding of multiple pathologies within aMCI and naMCI. Although there is a plethora of pathologies, naMCI had a propensity for an increased density of LBs and aMCI for increased NFTs in select anatomic regions. Furthermore, executive dysfunction in the setting of MCI was associated with the presence of CWMR and CAA.
